# Potential biomarkers of major depression diagnosis and chronicity

**DOI:** 10.1371/journal.pone.0257251

**Published:** 2021-09-29

**Authors:** Ana Cecília de Menezes Galvão, Raíssa Nobrega Almeida, Geovan Menezes de Sousa Júnior, Mário André Leocadio-Miguel, Fernanda Palhano-Fontes, Dráulio Barros de Araujo, Bruno Lobão-Soares, João Paulo Maia-de-Oliveira, Emerson Arcoverde Nunes, Jaime Eduardo Cecilio Hallak, Jerome Sarris, Nicole Leite Galvão-Coelho

**Affiliations:** 1 Laboratory of Hormone Measurement, Postgraduate Program in Psychobiology and Department of Physiology and Behavior, Federal University of Rio Grande do Norte, Natal, RN, Brazil; 2 Brain Institute, Federal University of Rio Grande do Norte, Natal, RN, Brazil; 3 National Institute of Science and Technology in Translational Medicine, Ribeirao Preto, Brazil; 4 Department of Biophysics and Pharmacology, Federal University of Rio Grande do Norte, Natal, RN, Brazil; 5 Department of Clinical Medicine, Federal University of Rio Grande do Norte, Natal, RN, Brazil; 6 Department of Psychiatry, Federal University of Rio Grande do Norte, Natal, RN, Brazil; 7 Department of Neurosciences and Behavior, University of Sao Paulo (USP), Ribeirão Preto, SP, Brazil; 8 NICM Health Research Institute, Western Sydney University, Westmead, Australia; 9 Professorial Unit, Department of Psychiatry, The Melbourne Clinic, University of Melbourne, Melbourne, Australia; National Institutes of Health, UNITED STATES

## Abstract

**Background:**

Molecular biomarkers are promising tools to be routinely used in clinical psychiatry. Among psychiatric diseases, major depression disorder (MDD) has gotten attention due to its growing prevalence and morbidity.

**Methods:**

We tested some peripheral molecular parameters such as serum mature Brain-Derived Neurotrophic Factor (mBDNF), plasma C-Reactive Protein (CRP), serum cortisol (SC), and the salivary Cortisol Awakening Response (CAR), as well as the Pittsburgh sleep quality inventory (PSQI), as part of a multibiomarker panel for potential use in MDD diagnosis and evaluation of disease’s chronicity using regression models, and ROC curve.

**Results:**

For diagnosis model, two groups were analyzed: patients in the first episode of major depression (MD: n = 30) and a healthy control (CG: n = 32). None of those diagnosis models tested had greater power than Hamilton Depression Rating Scale-6. For MDD chronicity, a group of patients with treatment-resistant major depression (TRD: n = 28) was tested across the MD group. The best chronicity model (p < 0.05) that discriminated between MD and TRD included four parameters, namely PSQI, CAR, SC, and mBDNF (AUC ROC = 0.99), with 96% of sensitivity and 93% of specificity.

**Conclusion:**

These results indicate that changes in specific biomarkers (CAR, SC, mBDNF and PSQI) have potential on the evaluation of MDD chronicity, but not for its diagnosis. Therefore, these findings can contribute for further studies aiming the development of a stronger model to be commercially available and used in psychiatry clinical practice.

## Introduction

The neurobiology of major depression disorder (MDD) is still a concern among physicians and scientists [1]. MDD is a multifactorial disorder with a complex pathophysiology: neither the biological changes are similar in all patients nor do they evolve with the same intensity [2–4]. Some studies show that patients with a first depressive episode or mild MDD have a greater salivary cortisol awakening response (CAR) and serum cortisol (SC) than healthy volunteers, while they show similar levels of mature brain-derived neurotrophic factor (mBDNF) and C-reactive protein (CRP) [5–7]. In contrast, patients with treatment-resistant depression (TRD) have often shown lower levels of SC and CAR, and higher levels of mBDNF and CRP, when compared to healthy controls [5, 8, 9]. Thus, the pathophysiology of MDD seems to somewhat rely on the chronicity of the disease [5, 10, 11].

Changes in sleep quality are also frequent in MDD. It integrates MDD diagnosis [12–14] and may be a predictor of treatment relapse [15]. While patients with mild MDD show weak sleep changes [5, 16], stronger disruptions are associated with TRD [5, 17, 18]. It is interesting to note that some of those biological changes associated with MDD are also related to sleep disorders [15, 19–21].

Recently, a massive research effort has been made toward the search for MDD biomarkers, which are measurable parameters that can indicate biological states and the response to ongoing treatments [22–24]. Specifically, the suitable use of biomarkers for mental disorders could provide support for a more precise diagnosis and prognosis, as well as for a better identification of clinical evolution [24, 25]. It could also be used as a complementary tool for choosing and monitoring treatments, thus helping to predict the occurrence of remission and relapse [1, 22, 23, 26].

Currently, there is a belief that probably no single biomarker *per se* can provide enough information to help in MDD diagnosis or support the investigation of its severity [27]. In this sense, a novel paradigm comprising a multimodal biomarker panel emerged in recent research. This panel of multiple biomarkers provides a more complete pathophysiological profile of patients, improving the chances of assisting the clinical practice in a more assertive way [22, 28, 29]. In this search for useful sets of biomarkers, a group of scientists developed the Research Domain Criteria (RDoC). It consists in a large American genomic and neuroscientific project that aims to identify distinct biomarkers for incidence risk, diagnose, and severity of several mental illness [30].

Like RDoC, most part of studies with plural molecular biomarkers panels for MDD are grounded on genetic [31, 32] or metabolomics approaches [33, 34]. There are few studies analyzing neuroendocrine-immune targets as part of a plural biomarker panel [35–37]. In contrast, these targets, such as cortisol and inflammatory cytokines, are the most investigated as single biomarkers of MDD [38–41]. Noteworthy, some of these biomarkers are already measured in routine exams, which facilitate their insertion into a wider panel that could be useful in clinical practice [42].

Therefore, considering the clinical demands for validation of a useful set of molecules and biological processes that can cooperate in MDD and may help in medical practice, in this work we proposed to test some specifics peripheral molecular parameters, such as CAR, SC, mBDNF and CRP, as well as the Pittsburgh sleep quality inventory (PSQI), as part of a multibiomarker panel for diagnosing and evaluating the chronicity of MDD. For this aim, we used regression model and the ROC curves. We hypothesized that the two types of cortisol measures (CAR and SC) can be assumed as a critical component in the proposed model for MDD diagnosis, while a larger panel with all tested molecular parameters and PSQI will have greater accuracy for identification of MDD chronicity.

## Methodology

### 1. Ethical aspects

This is a mathematical study that uses data from a study conducted at Federal University of Rio Grande do Norte (UFRN), between 2014 and 2018 [5]. The sample size of original study was determined by G*Power (version 3.1.9.4) [43]. All participants provided written informed consent and participated in the research voluntarily. The procedures of the study complied with the ethical standards of the relevant national and institutional committees for human experimentation and with the Declaration of Helsinki of 1975, revised in 2008. The study protocol was approved by University Hospital (HUOL) Human Research Ethics Committee (protocol No. 579,479) and UFRN Human Research Ethics Committee (protocol No. 2,628,202). The study was registered at http://clinicaltrials.gov (NCT02914769/U1111-1215-4472). All information used in this study was kept confidential.

### 2. Participants

The recruitment of participants was performed by advertising on local and social media, as well as via psychiatry referrals. A clinical screening by trained psychiatrists who used the Structured Clinical Interview for Axis I (DSM-IV) and the Hamilton Depression Rating Scale 17 (HAM-D 17) [44] was carried out with all volunteers for attending the inclusion and exclusion criteria.

**Major Depression patients (n = 58; 21 men and 37 women).** The general exclusion criteria for MDD patients were: present with a current diagnosis of drug abuse or substance-related disorder, schizophrenia, bipolar affective disorder, mania or hypomania and neurological disorder.

After screening the volunteers diagnosed with MDD were clustered into two groups:

1. Patients in first depressive episode (MD): A group with 30 participants newly diagnosed with MDD (14 men and 16 women), who never used antidepressants and during the study were free of medications with effects on cognition, mood, neurovegetative, immune and endocrine functions.
2. Patients with treatment-resistant depression (TRD): A group with 28 MDD patients (7 men and 21 women) who did not respond to at least two previous standard antidepressant pharmacotherapies and during the study underwent a 15-day washout period without antidepressants use. The washout is a procedure carried out when changes in antidepressant medication is needed.

**Healthy controls (CG: n = 32; 15 men and 17 women).** A group of healthy volunteers with similar socio-demographic characteristic of patients, and without diagnosis of physical, sleep, neurological or psychiatric disorders. Along this study they were also free of medications with effects on cognition, mood, neurovegetative, immune and endocrine functions.

For all participants, patients and controls, an additional inclusion criterion was being available to overnight in the University Hospital. In addition, for women, an additional exclusion criterion was not being pregnant or have given birth in last 6 months during the study period.

### 3. Experimental design

After screening, the volunteers were individually invited to overnight at University (UFRN), in order to collect their saliva at awakening to measure CAR. Therefore, on the following day, around 6:00 am, saliva samples were collected: 1st collection was performed at the volunteer’s awakening (T0); 2nd collection with 30 minutes after awakening (T30) and 3rd collection at 45 minutes after awakening (T45). It was followed by blood collection for dosage of cortisol, CRP and mBDNF. All volunteers were fasting for approximately 8 hours. For more details see Galvão et al. (2021).

### 4. Biochemical analysis

All biochemical dosages were blindly performed through ELISA technique, in duplicates. Salivary cortisol was measured by direct competitive ELISA using the DRG-SLV 4635 kit. Salivary CAR was calculated as the area under the curve (AUC) of the three saliva points collected at T0, T30 and T45 [45]. For dosage of serum cortisol we used the DRG 1887 kit (direct competitive ELISA). The serum mBDNF was dosed by SK00752-01 Aviscera bioscience ELISA kit (Human, Mouse, Rat sandwich ELISA). CRP was assessed by latex agglutination of EBRAM, which qualitatively indicates the presence or not of inflammation. In this study, the intra and inter-assay coefficients of variation (CV) were respectively 3.97 and 13.01% for serum cortisol, 4.78 and 16.30% for CAR, as well as 6.15 and 21% for mBDNF.

### 5. Psychometric instruments

The HAM-D 17 that was assessed on screening phase is widely used to MDD diagnosis and to quantify depressive symptoms [46].

The 6-item version of HAM-D (HAM-D 6) is a shorter form of HAMD-17 that has a one-dimensional structure composed by the core of symptoms of depression, such as depressed mood, feeling of guilt, work and activities, motor retardation, psychological anxiety, and somatic symptoms [47–49]. Currently, some mathematical model studies of MDD biomarkers have used the HAM-D 6 in their investigations [10, 50], since its one-dimensional feature is easier to mathematically explorate than the multidimensional HAM-D 17. Therefore, in the regression models explored in this study, the HAM-D 6 was chosen as the standard, with which the predictive value of the potential biomarkers’ models were compared.

The Pittsburgh Sleep Quality Index (PSQI) is a self-reported instrument used to assess sleep quality and sleep disturbances over a 1-month time interval [51, 52]. This tool has an overall score ranging from 0 to 21 points, which can be categorized into good sleep (0–4 points), poor sleep (5–10 points), and sleep disorder (greater than 10 points).

### 6. Statistical analysis

The groups (MD, CG and TRD) were the categorical independent variables in this study. The molecular parameters (CAR, SC, mBDNF), total PSQI score and HAM-D 6 score were the continuous quantitative dependent variables, and the CRP, a categorical dependent variable (positive/ negative indicator of systemic inflammation). CAR, serum cortisol (SC) and total PSQI were log-transformed to reach Gaussian distribution. To explore the clinical and sociodemographic characteristics between depressive groups, we applied the Mann-Whitney and independent t-test.

First, we used the Boruta random forest-based algorithm to rank sociodemographic characteristics, BMI, and dependent variables with respect to their relevance to discriminate the groups. Those variables scored above the shuffled data (I > 2.98) were categorized as relevant [53] and used to build the regression models to predict the diagnosis and MDD chronicity.

An aim of this study is having as result a model with true clinical value. Thus, on exploration of MDD diagnosis we only tested the dependent variables for CG *vs* MD, since the clinical diagnosis is potentially more complex to operationalize for subjects with mild than for patients affected by severe symptoms. For models of MDD chronicity, we tested the dependent variables for MD and TRD groups, which in addition to show a significantly difference on the average severity of depressive symptoms (HAM-D 17), had consistent differences in the disease duration, number of episodes, and number of previous treatments.

The regression models were made using the Generalized Linear Mixed Model by *glmmTMB* package [54]. Each model had from 1 to 6 dependent variables with multiple distinct combinations of molecular biomarkers (CAR, SC, mBDNF and CRP), PSQI and the HAM-D 6 score. Sex and age were controlled in all models, that is, they were used as covariates. The best model must have the lowest Akaike Information Criterion (AICc) value and the delta AICc minor than 2. This selection was performed through the *dredge* function of the *MuMIn* package [55].

Then, the Receiver Operating Characteristic Curve (ROC) was applied to test the accuracy of the best regression model [56–59]. The ideal and maximum AUC value of the ROC curve to group discrimination is 1, and values minor than or equal to 0.5 are not significant for group discrimination. It was established that an AUC value equal or larger than 0.8 was necessary for the model to be classified as “good” [60–62]. The model sensitivity was accessed by the probability of the model to show a positive diagnosis in an individual affected by a disease, while specificity through the probability that the model shows a negative result in an individual without the disease [63]. This analysis was performed with the *pROC* package.

All statistical analyzes were performed using the RStudio program. The significance level considered was p ≤ 0.05 in all tests.

## Results

After the screening of 640 volunteers, 58 MDD patients and 32 healthy controls were admitted in this study. The consolidated standards for clinical trial reports (CONSORT) can be found in the supplementary material, S1 Fig. Clinical and sociodemographic characteristics of participants by group (CG, MD, and TRD) are in S1 Table. All groups had a larger proportion of women than men (CG = 53.12%, MD = 53.33% and TRD = 75%). The average age (in years) of groups were: CG μ = 27.06 ± 6.42, MD μ = 24.2 ± 3.84 and TRD μ = 41.57 ± 11.61. All groups had most part of volunteers with low income and undergraduate education. BMI was similar between groups (Mann-Whitney; U = 310 p = 0.08) (S1 Table). The Boruta algorithm showed that from these clinical and sociodemographic characteristics, age is the most relevant for discrimination of MD and CG (I = 5.89) as well as MD and TRD (I = 35.02), therefore it was included as covariate in all predictive models of diagnose and chronicity tested (S2 Fig). As many studies have shown a sex dimorphism in MDD diagnosis in favor of woman [64], and our sample was predominantly women, sex was also included as covariate in all mathematical models analyzed.

The MD group presented in average mild depressive symptoms, while the TRD showed severe symptom levels (HAM-D 17: MD μ = 12.56 ± 0.56, and TRD μ = 21.57 ± 0.99; Mann-Whitney: U = 34.5 p < 0.001). TRD had worse sleep quality than MD (t independent; t = 4.34 p < 0.001). Some comorbidities were diagnosed in TRD participants, such as personality (Histrionic: n = 10/50%; Borderline: n = 9/45%; Schizoid: n = 1/5%) and anxiety disorder (Generalized Anxiety Disorder [GAD]: n = 10/83.33%; Panic Disorder [PD]: n = 5/17.24; Social Phobia [SP]: n = 2/16.67%). However, these comorbidities did not modulate biomarkers’ levels, as shown in Galvão et al (2021); therefore, they were not included as covariates in the tested predictive models.

### MDD diagnosis

**Fig 1:**
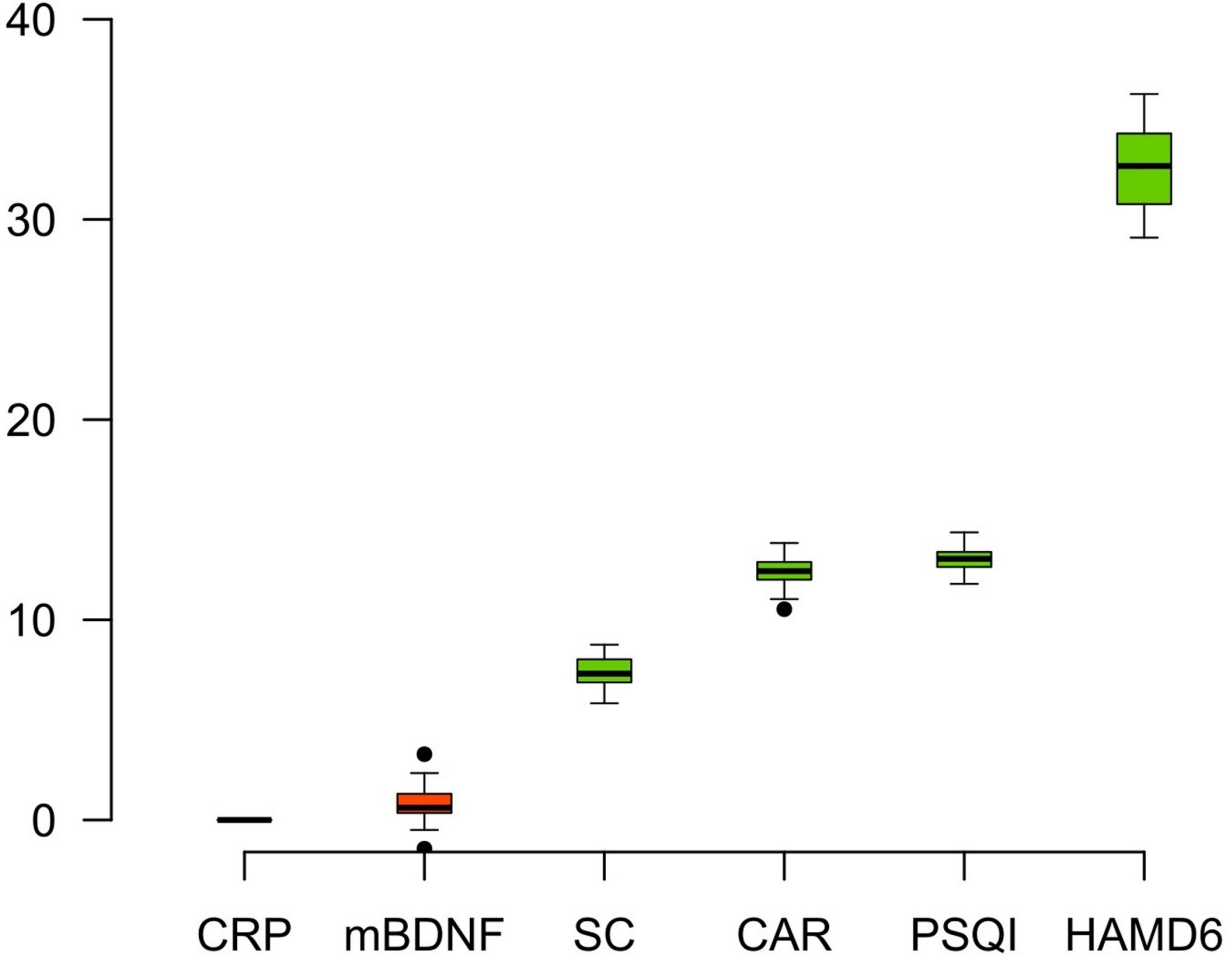
The random forest-based algorithm (Boruta) of parameter relevance for discrimination between patients in first depressive episode (MD n = 30) and control group (CG n = 32). Colors: green = relevant (p<0.05); yellow = tentative of relevance; red = no relevant.

The variables selected by Boruta test for the discrimination of MD (n = 30) and CG (n = 32) were: HAM-D 6 (I = 32.60), PSQI (I = 13.03), CAR (I = 12.32) and SC (I = 7.43) (Fig 1). The mBDNF (I = 0.86) and CRP (I = 0) were not significant to discriminate between these groups.

**Fig 2:**
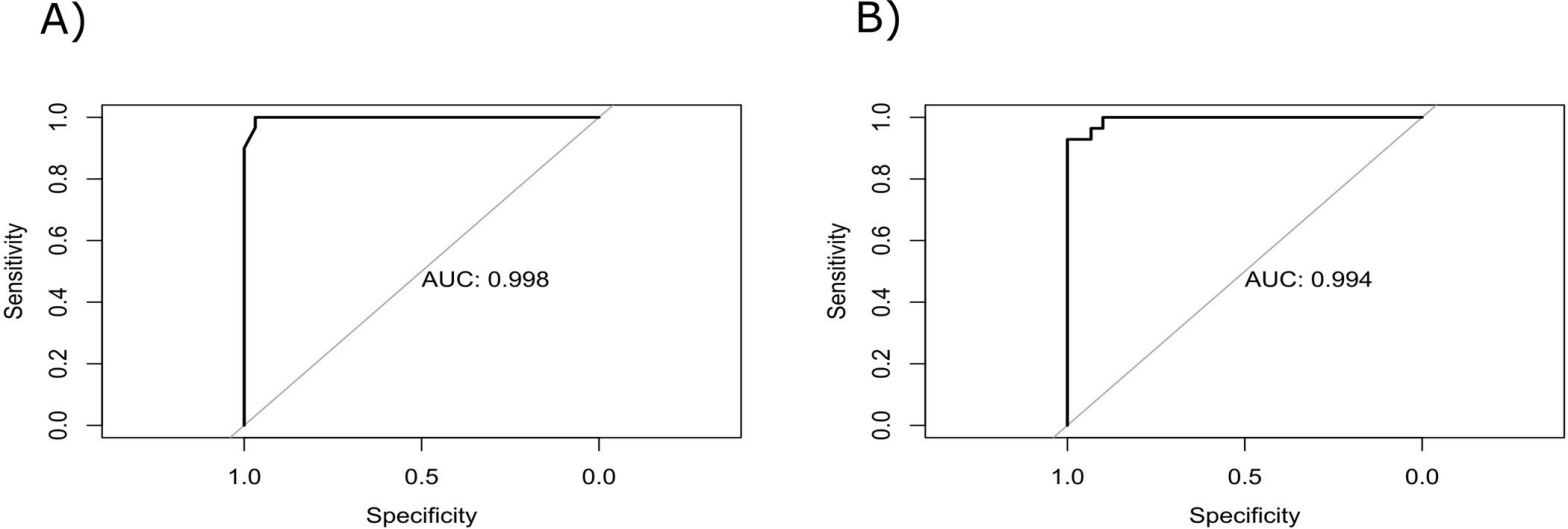
Area under the curve (AUC) of Receiver Operating Characteristic (ROC) curve for: A) Regression model for diagnosis of patients with first episode of major depression from healthy controls. B) Regression model for major depression disorder chronicity, for discrimination between patients in first depressive episode and patients with treatment-resistant depression.

Then, the regression models for MDD diagnosis made of multiple combinations of HAM-D 6, PSQI, CAR and SC resulted in four statistically significant models with **Δ**AICc < 2 (Table 1). However, from these models, those containing biomarkers were not stronger than HAM-D 6 for diagnosis of *de novo* patients. Therefore, the best regression model for MDD diagnosis found in this study included only HAM-D 6 (AICc = -37.48, B = 0.06, Z = 16.29, p < 0.001) (Table 1). In our sample, this model showed 100% of sensitivity and 96% of specificity (AUC ROC = 0.99) (Fig 2A).

**Table 1:** Regression models for major depression disorder diagnosis, possible predictive models of discrimination between patients in first depressive episode (MD: n = 30) and the healthy controls (CG: n = 32).

Models	AICc	ΔAICc	Β	z-score	p-value
**I**					
**HAM-D 6**	**-37.48**	**0**	**0.06**	**16.29**	**< 0.001**
**II**					
**HAM-D 6**	-36.76	0.72	0.06	16.29	< 0.001
**CAR**			0.07	1.29	0.19
**III**					
**HAM-D 6**	-36.46	1.02	0.06	16.29	< 0.001
**PSQI**			0.007	1.16	0.24
**IV**					
**HAM-D 6**	-35.73	1.75	0.06	16.29	< 0.001
**CAR**			0.07	1.29	0.19
**PSQI**			0.007	1.16	0.24
**V**					
**HAM-D 6**	-35.42	2.05	0.06	16.29	< 0.001
**SC**			0.04	0.47	0.63
**VI**					
**HAM-D 6**	-34.29	3.19	0.06	16.29	< 0.001
**CAR**			0.07	1.29	0.19
**SC**			0.04	0.47	0.63
**VII**					
**HAM-D 6**	-34.27	3.20	0.06	16.29	< 0.001
**SC**			0.04	0.47	0.63
**PSQI**			0.007	1.16	0.24
**VIII**					
**HAM-D 6**	-33.15	4.32	0.06	16.29	< 0.001
**CAR**			0.07	1.29	0.19
**SC**			0.04	0.47	0.63
**PSQI**			0.007	1.16	0.24

Bold result indicates the best regression model with the lowest AICc and **Δ**AICc< 2. All models showed in this table were statistically significant (p< 0.05) and controlled by age and sex, while four of them had **Δ**AICc< 2. CAR: cortisol awakening response; SC: serum cortisol; PSQI: Pittsburgh sleep quality index; HAM-D 6: Hamilton Depression Rating Scale with 6 items.

### MDD chronicity

**Fig 3:**
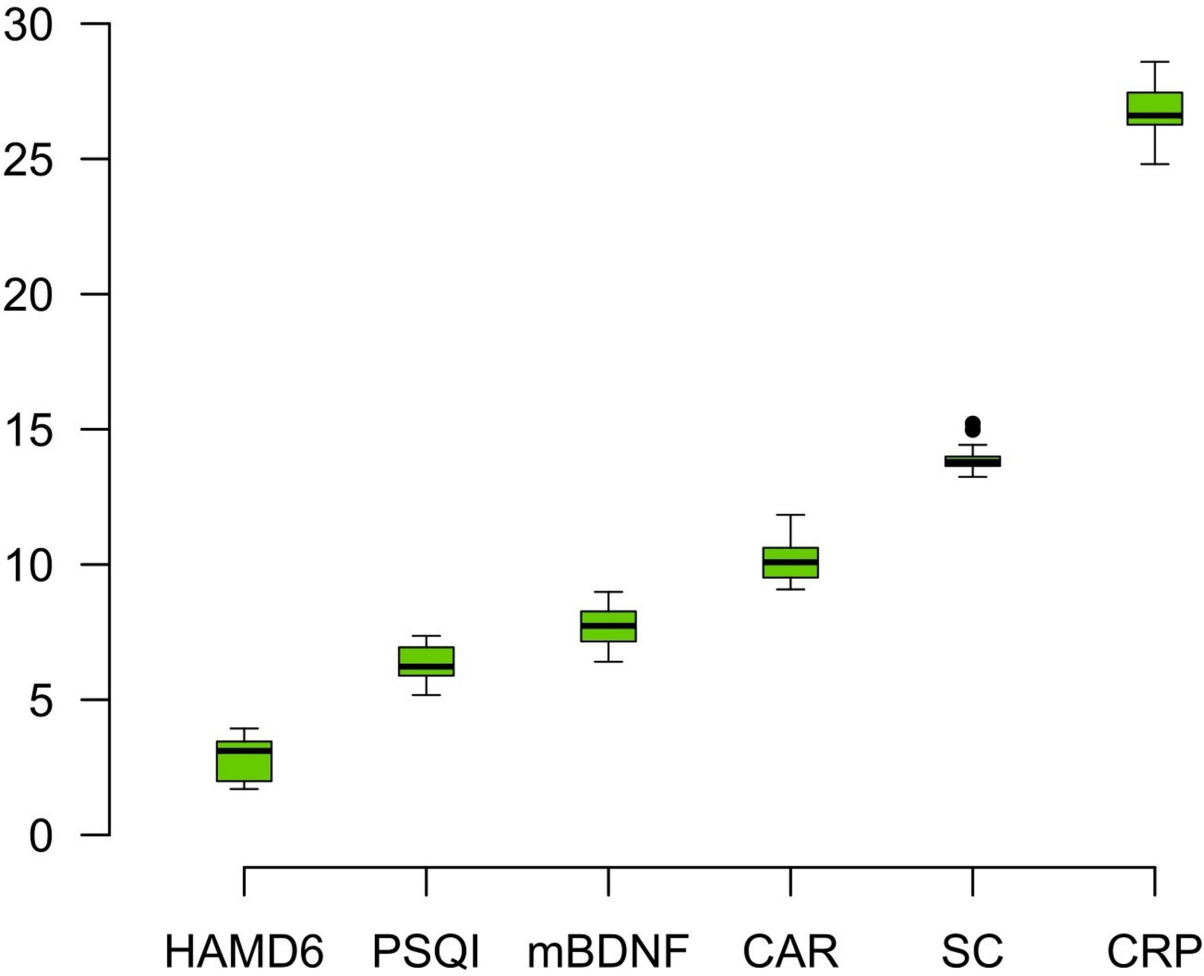
The random forest-based algorithm (Boruta) of parameter relevance for discrimination between patients in first depressive episode (MD n = 30) and patients with treatment-resistant depression (TRD n = 28). Colors: green = relevant (p<0.05); yellow = tentative of relevance; red = no relevant.

The variables selected by Boruta test for discrimination of MD and TRD (n = 28) were: CRP (I = 26.89), SC (I = 13.96), CAR (I = 10.26), mBDNF (I = 7.96), PSQI (I = 6.28) and HAM-D 6 (I = 2.98) (Fig 3).

The mathematical models for MDD chronicity made of all variables previously select by Boruta algorithm resulted in two statistically significant regression models with **Δ**AICc < 2 (Table 2). The best regression model for MDD chronicity included (AICc = 32.57): CAR (B < 0.001, Z = 2.70, p = 0.006), SC (B < 0.001, Z = 4.46, p < 0.001), mBDNF (B < 0.001 Z = 2.31 p = 0.02) and PSQI (B < 0.001, Z = 2.06, p = 0.03). The model has 96% of sensitivity and 93% of specificity (AUC ROC = 0.99) (Fig 2B).

**Table 2:** Regression models for major depression disorder chronicity, possible predictive models of discrimination between patients in first depressive episode (MD: n = 30) and patients with treatment-resistant depression (TRD: n = 28).

Models	AICc	ΔAICc	Β	z-score	p-value
**I**					
**CAR**	**32.57**	**0**	**< 0.001**	**2.70**	**0.006**
**SC**			**< 0.001**	**4.46**	**< 0.001**
**mBDNF**			**< 0.001**	**2.31**	**0.02**
**PSQI**			**< 0.001**	**2.06**	**0.03**
**II**					
**CAR**	34.37	1.80	< 0.001	2.70	0.006
**SC**			< 0.001	4.46	< 0.001
**mBDNF**			< 0.001	2.31	0.02
**III**					
**CAR**	35.31	2.74	< 0.001	2.70	0.006
**SC**			< 0.001	4.46	< 0.001
**PSQI**			< 0.001	2.06	0.03
**IV**					
**SC**	37.73	5.16	< 0.001	4.46	< 0.001
**mBDNF**			< 0.001	2.31	0.02
**PSQI**			< 0.001	2.06	0.03
**V**					
**CAR**	38.06	5.49	< 0.001	2.70	0.006
**SC**			< 0.001	4.46	< 0.001
**VI**					
**SC**	39.94	7.37	< 0.001	4.46	< 0.001
**PSQI**			< 0.001	2.06	0.03
**VII**					
**SC**	42.39	9.82	< 0.001	4.46	< 0.001
**mBDNF**			< 0.001	2.31	0.02
**VIII**					
**SC**	45.82	9.82	< 0.001	4.46	< 0.001
**IX**					
**CAR**	49.39	16.82	< 0.001	2.70	0.006
**mBDNF**			< 0.001	2.31	0.02
**PSQI**			< 0.001	2.06	0.03
**X**					
**CAR**	52.19	19.62	< 0.001	2.70	0.006
**mBDNF**			< 0.001	2.31	0.02

Bold result indicates the best regression model with the lowest AICc and ΔAICc< 2. All models showed in this table were statistically significant (p< 0.05) and controlled by age and sex, while 2 of them were ΔAICc< 2. CAR: cortisol awakening response; SC: total serum cortisol; mBDNF: mature brain-derived neurotrophic factor; PSQI: Pittsburgh sleep quality index.

## Discussion

In this work, we searched for mathematical models made of a multibiomarker panel for a potential use in diagnosis and evaluation of MDD chronicity. When we searched for a possible model for MDD diagnosis, those models made of potential biomarkers were not stronger than HAM-D 6 for discrimination of *de novo* patients from healthy controls. Therefore, the HAM-D 6 still fitted as the best strategy for MDD diagnosis, with 100% of sensibility and 96% of specificity. On the other hand, for MDD chronicity, the best model included a mixed panel made of serum cortisol, salivary cortisol awakening response, serum mature BDNF and the total score of PSQI scale. This panel showed 96% of sensitivity and 93% of specificity to discrimination of TRD from MD patients.

Currently, cortisol is pointed as a good MDD biomarker. However, our model partially contradicts this view since our best diagnosis model doesn’t include this hormone. Although changes in cortisol are frequent in MDD [65, 66], we must consider that some of these changes have small effect sizes and large variance [26, 67, 68], mainly in newly diagnosed patients [5, 6, 69].

However, some studies that used a mathematical prediction model pointed to serum cortisol as a critical biomarker of MDD diagnosis [38, 39]. Nevertheless, it is important to highlight that the sample of those studies did not comprise *de novo* patients, but participants with distinct MDD severities jointed into a single group [38, 39], in contrast to our sample. Considering that a MDD diagnosis is more complex for subjects with mild than it is for severely impaired patients, our model contemplates a sample with typical characteristics for a first MDD diagnosis and thus can be especially useful for clinical purposes. Therefore, our results indicate that cortisol changes have not pivotal value for being used as a complement tool to define first MDD diagnosis, and the HAM-D 6 seems enough to provide it.

On the other side of investigation, for MDD chronicity, both cortisol measures: salivary cortisol awakening response and serum cortisol, as well as serum mBDNF and sleep quality (PSQI), were part of the best predictive model. Similar to what we have found, an impairment on HPA axis function was associated to severity of depression in a study of mathematical prediction [8].

As we had hypothesized, the levels of serum mBDNF were included in the best model of MDD chronicity. Despite there is not a consensus about BDNF changes in MDD, some studies have suggested its reduction in antidepressant drug-free patients when compared to healthy subjects [5, 70, 71], while others suggest an increased BDNF levels in treated patients that can be partially resulted from previous antidepressant treatments [5, 72, 73]. Therefore, this difference between *de novo* and treatment-resistant patients with major depression had made this biomarker important in the evaluation of MDD chronicity.

The inclusion of PSQI in the proposed model for MDD chronicity confirms the importance of impairments in sleep quality in the evolution of MDD. Frequently, changes in sleep quality get worse along the course of the disease [46, 74]. Stronger sleep disturbances are related with more severe MDD symptoms and worse treatment response [18]. It is pertinent that the HPA axis and BDNF are often related with sleep disturbances [17, 22, 75, 76].

In contrast to our initial hypothesis, CRP was not part of the model that best fitted for assessing MDD chronicity. While changes in inflammation are often lacking in *de novo* patients [6, 77], a mild and chronic inflammatory profile is observed in more severe MDD patients [78, 79]. A study that exanimated different molecular biomarkers along MDD chronicity found for both males and females an association in CRP levels and number of MDD episodes [80, 81]. Probably the MDD chronicity model of this study did not include CRP because this biomarker was used here as a qualitative measure, which is lesser sensible than a quantitative value. Therefore, future studies using it as a quantitative data are encouraged.

Therefore, this model of MDD chronicity can be useful for a better understanding of its neurobiology and in the future help in medical decisions about which biological pathway(s) should be targeted to improve treatments. For instance, treatments aiming to regulate cortisol levels might be considered for treatment-resistant patients. The several antidepressants currently used may have distinct actions on the HPA axis. Moreover, the antidepressant treatment duration has a large impact on the modulation of HPA axis as well [82, 83]. Since cortisol is a hormone with multiple roles, its return to homeostatic levels would probably leads to improvements in immune function, neuroplasticity process, and sleep quality [18, 79, 84]. All these are biological processes usually impaired in MDD, most especially in those with severe symptoms [15, 85, 86].

Despite not all patients with major depression progress to TRD—in average 30% of them shows recurrent and/or chronic MDD—this feature is associated to high morbidity and leads to great harm. TRD patients have shown large disability in individual, social, and work fields, and it can ultimately increase suicide risk [23, 64]. For instance, the TRD patients of our sample had about 10 years of MDD, with some volunteers showing until 20 years of disease. Therefore, despite only one-third of MDD patient becoming TRD, studies with this group of patients are important due to its massive damages. Then, a mathematical model like this one could help in understanding the psychobiological ground behind the disorder and in clinical practice.

Moreover, a point that we must highlight in favor of our models is that the studies focusing on biomarkers models for MDD usually did not include psychometric instruments to measure depressive symptoms, such as the HAM-D, as we did [35, 37, 38, 87]. In this sense, it is pointed out that only a model with a robust power, that is larger than the most used psychometrics tools, such as HAM-D, justifies its clinical applicability [87]. Another relevant aspect of our exploratory models was that we controlled both sex and age when analyzing those biomarkers, since many studies have pointed to a possible modulation of molecular biomarkers by these two variables [88–91].

Nevertheless, this study presented some limitations. Our sample has a restricted size and severity levels, and, as a result of the inclusion/exclusion criteria, it may not represent the real profile of the disorder among our population. Moreover, we did not perform a cross-validation analysis using another dataset to confirm our findings and establish a cutoff point for the biomarkers to distinguish them between groups in chronicity model.

Though, our results show the relevance of testing potential biomarkers of MDD in statistical models of adequate prediction [46] and bring a step-forward showing that only for MDD chronicity, and not for diagnosis of *de novo* patients, some of those biomarkers are somewhat more efficient than the HAM-6, namely: salivary cortisol awakening response and serum cortisol, as well as serum mBDNF and sleep quality (PSQI). Consequently, further studies of cross-validation analysis with larger and heterogeneous populations should be done to verify the proposed model of MDD chronicity and establish the biomarkers’ cutoff, then a robustly validated model could be commercially available to be used in psychiatry clinical practice to assist in charting MDD clinical stages, as well as in choosing the best treatment for patients.

## Supporting information

S1 Fig Flow diagram of the consolidated standards for clinical trial reports (CONSORT). CG: control group, PG: patient group, MD: patients in first depressive episode, TRD: patients with treatment-resistant depression.
(DOCX)

S2 Fig Importance of sociodemographic characteristics and BMI for the discrimination of groups. Random forest-based algorithm (Boruta): A) Patients with first episode of major depression (MD, n = 30) and control group (CG, n = 32). B) MD and Patients with treatment-resistant major depression (TRD, n = 28). Colors: green = relevant characteristic; yellow = tentative of relevance; red = no relevant characteristic; blue = randomly shuffled data at a maximum, mean and minimum level.
(TIF)

S1 Table Clinical and sociodemographic characteristics of participants. (DOCX)
