# Supplementary material for: Potential biomarkers of major depression diagnosis and chronicity
Source: PLoS One. 2021 Sep 29;16(9):e0257251. doi: 10.1371/journal.pone.0257251 (PMC8480905; doi:10.1371/journal.pone.0257251)
Supplement: S1 Fig — CG: control group, PG: patient group, MD: patients in first depressive episode, TRD: patients with treatment-resistant depression. (DOCX) [file pone.0257251.s001.docx]

Assessed for eligibility (n = 640)

Allocated

(n = 90)

Excluded (n = 550)

- Excluded pre-screening (n = 50)

- Not meeting inclusion criteria (n = 268)

- Refused to participate (n = 158)

- Others reasons (n = 74)

Allocated to CG (n = 32)

Allocated to PG (n = 58)

Analyzed to MD (n = 30)

**Enrollment:**

**Allocation:**

**Analysis:**

Analyzed to TRD (n = 28)

Analyzed to CG (n = 32)
