# Supplementary figures and images for: Potential biomarkers of major depression diagnosis and chronicity
Source: PLoS One. 2021 Sep 29;16(9):e0257251. doi: 10.1371/journal.pone.0257251 (PMC8480905; doi:10.1371/journal.pone.0257251)

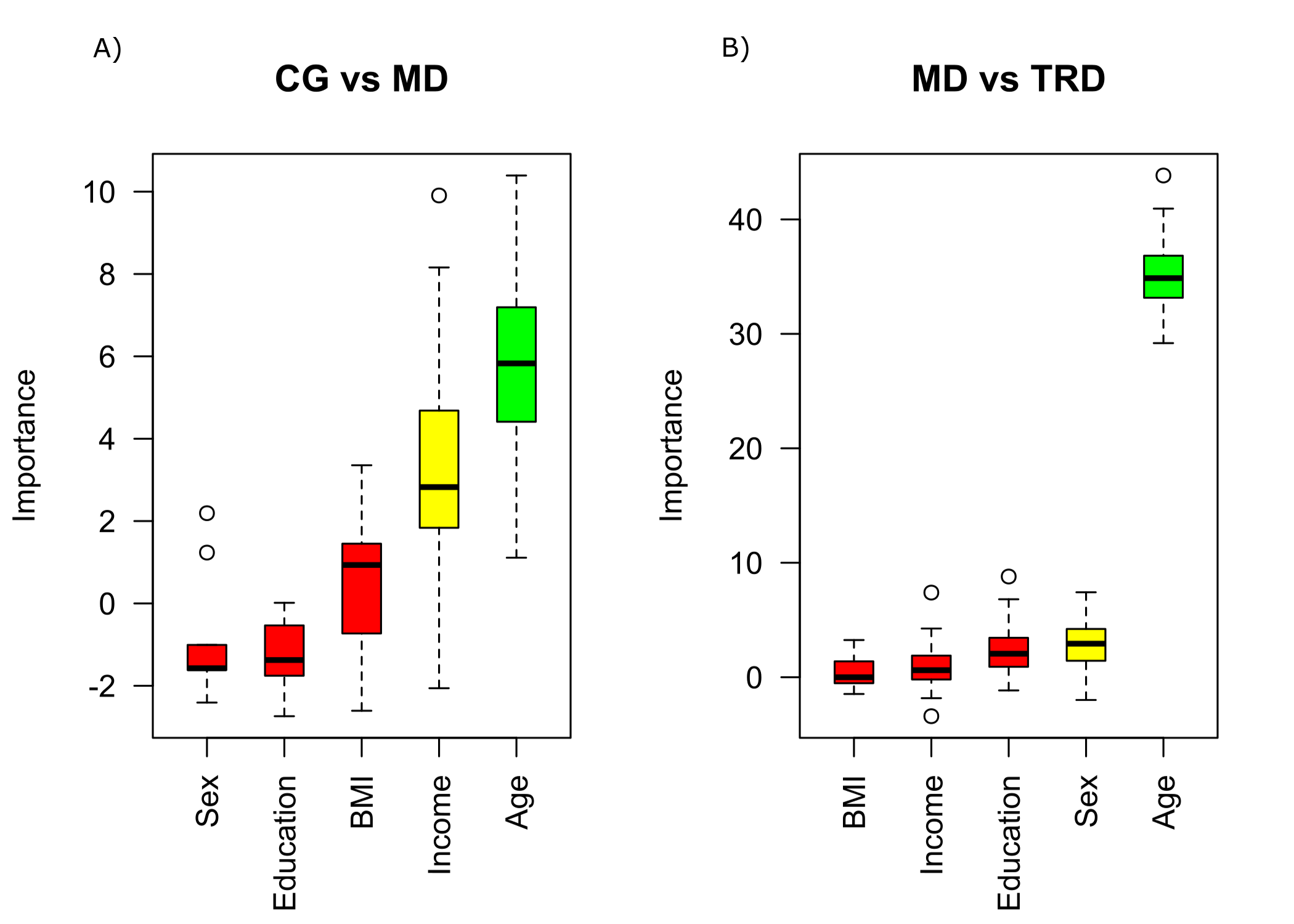

Supplement: S2 Fig — Random forest-based algorithm (Boruta): A) Patients with first episode of major depression (MD, n = 30) and control group (CG, n = 32). B) MD and Patients with treatment-resistant major depression (TRD, n = 28). Colors: green = relevant characteristic; yellow = tentative of relevance; red = no relevant characteristic; blue = randomly shuffled data at a maximum, mean and minimum level. (TIF) [file pone.0257251.s002.tif]
