# Supplementary material for: Potential biomarkers of major depression diagnosis and chronicity
Source: PLoS One. 2021 Sep 29;16(9):e0257251. doi: 10.1371/journal.pone.0257251 (PMC8480905; doi:10.1371/journal.pone.0257251)
Supplement: S1 Table — (DOCX) [file pone.0257251.s003.docx]

|  |  | CG | MD | TRD |
| --- | --- | --- | --- | --- |
| Sample (n) | | 32 | 30 | 28 |
| Gender | Women | 17 (53.12%) | 16 (53.33%) | 21 (75%) |
|  | Men | 15 (46.88%) | 14 (46.67%) | 7 (25%) |
| Age (years) | | μ = 27.06 ± 6.43 | μ = 24 ± 3.85 | μ = 41.57 ± 11.61 |
| Education | Undergraduate | 17 (53.13%) | 18 (60%) | 22 (78.57%) |
|  | Graduate | 15 (46.87%) | 12 (40%) | 6 (21.43%) |
| Familiar Income | Low | 18 (56.25%) | 23 (76.67%) | 23 (82.14%) |
|  | Medium | 14 (43.75%) | 7 (23.33%) | 5 (17.86%) |
|  | High | 0 (0%) | 0 (0%) | 0 (0%) |
| Clinical Aspects | HAM-D 17 | μ = 0.69 ± 1.15 | μ = 12 ± 3.11 | μ = 21.57 ± 5.27 |
|  | Duration of disease | 0 | < 30 days | μ = 10.71 ± 9.72 |
|  | Episodes | 0 | 1 | μ = 12.71 ± 14.74 |
|  | Number of previous treatment | 0 | 0 | μ = 2.71 ± 0.85 |
| BMI (kg/m²) | | μ = 23.98 ± 4.79 | μ = 25.49 ± 5.41 | μ = 27.62 ± 5.67 |
| PSQI | | μ = 5.66 ± 2.97 | μ = 9 ± 3.48 | μ = 14 ± 3.71 |

CG: control group; MD: patients in first depressive episode; TRD: patients with treatment-resistant depression; HAM-D: Hamilton Depression Rating Scale; BMI: body mass index; PSQI: Pittsburgh Sleep Quality Index.
